# Impact of positive surgical margin location after radical prostatectomy: a network meta-analysis

**DOI:** 10.1007/s00345-025-05479-7

**Published:** 2025-02-22

**Authors:** Athul John, Thomas Milton, Aashray Gupta, Mau T. Nguyen, Brandon Stretton, Joseph Hewitt, James Virgin, Joshua Kovoor, Rick Catterwell, Luke Selth, Michael O. Callaghan

**Affiliations:** 1 Adelaide Medical School, The University of Adelaide Faculty of Health and Medical Sciences, Adelaide, South Australia Australia; 2 Urology, Central Adelaide Local Health Network, Adelaide, South Australia Australia; 3 Urology, Southern Adelaide Local Health Network, South Australia Prostate Cancer Clinical Outcomes Collaborative, Adelaide, South Australia Australia; 4 College of Medicine and Public Health, Flinders Health and Medical Research Institute, Flinders University, Bedford Park, SA 5042 Australia; 5 Freemasons Centre for Male Health and Wellbeing, Flinders University, Bedford Park, SA 5042 Australia

**Keywords:** Positive surgical margin location, Prostate cancer, Radical prostatectomy, Biochemical recurrence, Positive surgical margin

## Abstract

**Objective:**

To perform a network meta-analysis comparing the impact of different positive surgical margin locations (Comparisons and intervention) on biochemical recurrence (Outcome) in patients undergoing radical prostatectomy (Population).

**Methods:**

According to the Preferred Reporting Items for Systematic Reviews and Meta-analysis (PRISMA) guidelines, a protocol was registered (PROSPERO: CRD42022119025) and a search across four databases was conducted (the MEDLINE, Scopus, Embase and Cochrane). The primary outcome was biochemical recurrence (BCR). A network meta-analysis was conducted. Further subgroup analysis was performed to evaluate studies exploring robot-assisted radical prostatectomy (RALP).

**Results:**

Our search yielded 1249 unique results; 22 studies were analysed. Anterior margins had the highest risk of BCR (HR 2.46, 95%CI 1.67–3.61, I^2^ = 76%) followed by posterior (HR 2.29, 95%CI 1.43–3.66, I^2^ = 0%), bladder base (HR 2.06, 95%CI 1.61–2.64, I^2^ = 69%), apical (HR 1.88, 95%CI 1.51–2.35, I^2^ = 59%), and posterolateral margins (HR 1.70, 95%CI 1.14–2.25, I^2^ = 60%). Given significant heterogeneity, subgroup analysis was performed. In the RALP subgroup, anterior margins also demonstrated the highest recurrence risk (HR 3.74, 95%CI 2.47–5.66, I^2^ = 0%), followed by apical (HR 2.43, 95%CI 1.97–8.00, I^2^ = 0%), posterior (HR 2.23, 95%CI 1.47–3.38), base (HR 1.65, 95%CI 1.29–2.11, I^2^ = 0%), and posterolateral margin (HR 1.54, 95%CI 1.07–2.22).

**Conclusions:**

The risk of BCR after radical prostatectomy varies by PSM location, with the highest recurrence risk observed at anterior margins.

**Supplementary Information:**

The online version contains supplementary material available at 10.1007/s00345-025-05479-7.

## Introduction

Prostate cancer accounts for 22% of all new male cancer diagnoses [1]. For localised prostate cancer, radical prostatectomy (RP) is one of the main curative treatments. Although curative, the surgery can have a significant impact on functional outcomes including incontinence and erectile dysfunction [1]. The degree of resection is a delicate balance between oncological and functional outcomes determined by the operating surgeon and disease biology. Positive surgical margins (PSM) are reported in 11–40% of men who have undergone RP [2]. The clinical significance of PSM after RP remains controversial since only 27–44% develop a biochemical recurrence (BCR), 6.8–24.3% develop systemic progression and 0.8–3.7% experience prostate cancer-related mortality over a 7–13 year follow-up period [3–6]. Further methods to stratify the risk of progression in these patients are warranted so that secondary treatments can be initiated appropriately and more appropriate surveillance can be pursued. This will ensure that resources are allocated to those at risk while sparing low-risk patients from compromised functional outcomes [7].

Over the past decade, various studies have attempted to subclassify histopathological features of PSM to predict the risk of BCR more accurately. One of the pathological variables that has been explored is the location of the PSM. Various comparisons have been made between anterior, posterior, bladder base, posterolateral, apical PSM and some soft tissue PSMs, all of which have been suggested to confer a higher risk of biochemical recurrence compared to NSM [8–10]. Despite these findings, there are no reviews or meta-analyses exploring this important histopathological variable. Given the lack of consolidated evidence, we aimed to conduct a systematic review and network meta-analysis answering the clinical question: does the location of PSM (Exposure and comparison) influence the risk of BCR (Outcome) after RP (Population)?

## Methods

### Search strategy

A systematic search was conducted using the MEDLINE, Scopus, Embase and Cochrane databases, including studies published up to 31st March 2023. A further literature search was performed by examining reference lists of studies identified in the database searches. The protocol was registered at the international prospective register of the systematic reviews database (PROSPERO: CRD42022119025). Search terms were identified and adjusted to match the requirements of each database with the assistance of a librarian (See Appendix).

### Inclusion criteria

Studies comparing the locations of PSM after RP in men with prostate cancer in predicting BCR or oncological outcomes were included (Table 1). The review followed the Preferred Reporting Items for Systematic Review and Meta-analysis Methods–Network meta-analysis (PRISMA—NMA) protocol [11]. The search results were independently reviewed by four authors, initially based on title and abstract screening (AJ, BS and TM) followed by a full-text review (AJ, JH, TM). Data extraction (AJ, AG) and risk of bias (JV and AJ) were conducted by two independent authors. Key reasons for exclusion included an absence of reporting on the location of PSM, abstract-only studies, duplicate study population and an absence of multivariable analysis.

**Table 1:** Summary of included studies

Study	Analysis group, Cohort type	PSM events/overall	Follow up—Median/Mean (Years)	Type of procedure	Gleason score included	Stage included	LN involvement	Adjuvant	BCR definition	Variables adjusted
Hsu et al. [19]	Apex v Base/Anterior, R	117/789	5.3(Mean)	Open	GG1/GG2/GG3/ > GG4 2.6%/30%/41%/26%	pT2/T3a/T3b/T4 (15.4%/43.6%/30.8%/10.2%)	pN1 21.4%	Excluded	> 0.2 ng/ml	PS, TV, LN, Gleason, LN, PSA, PSM Length, PSM location, stage
Dev et al. [5]	NSM v Apex/Base/Anterior/posterior, Base v Apex/Posterolateral/Ant, R	486/4000	6.2	Robot	GG1/GG2/GG3/ > GG4%(27.2%/38.9%/16%/17.9%)	pT2/T3a/ > T3b (45.9%/35.1%/19%)	23.9% of PSM	Excluded	2 readings > 0.2 ng/ml	A, PSA,BMI, Stage, Gleason, PS
Sammon et al. [34]	NSM v Apex/Bneck/Ant/Posterolateral, R	162/794	4.5	Perineal RP	6/7/8/9 29%/62.3%/3.7%/4.9%	pT2/3a/b 41.4%/47.5%/11.1%	Not performed	Excluded (11% received neoadjuvant)	2 readings > 0.2 ng/ml	A, PSA, Stage, Gleason, PS
Sooriakumaran et. al [25]	NSM v Apex/anterior/posterolateral/base Postlat v apex/base/ant	189/904	min 5 years	Robot only	GG1/GG2/GG3/ > GG4 40.4%/36.7%/13.3%/9.6%	pT2/pT3a/pT3b (9.4%/23.2%/60.5%)	NR	Excluded	1 reading > 0.2	PSA, Stage, Gleason, stage, Margin
Choo et [35]	NSM v Apex/Base, R	462/1874	3.6	Robot/Open (33.3%/66.7%)	6/7/8–10 (27%/66.7%/6.3%)	pT2ab/c/pT3a (11.6%/32.8%/55.5%)	0%	Excluded	> 0.2 ng/ml	Gleason, PSA, Stage, Margin
Chung et al. [36]	NSM v Base, R	50/368	2.1 (Mean)	Robot/Open (64%/36%)	6/7/8–10 (34.8%/49.5%/15.7%)	pT2/pT3 (39%/61%)	0%	Excluded	> 0.2 ng/ml	Gleason, PSA, stage, TV
Eastham et al. [10]	NSM v Apex/Base/Anterior/Posterior/Posterolateral	275/2442	2.9	Open	GG1/GG2/GG3/ > GG4) (50%/33%/11.2%/6%)	pT1ab/T1c/T2a/b/c/T3 (4%/49%/18%/14%/10%/4%)	5%	2%. Adjusted in analysis	> 0.2 ng/ml	PSA, Gleason, LN, Stage
Kordan et al. [37]	NSM v Apex, R	372/1667	1.75	Robot/Open (NA)	6/7/8–10 (36%/52%/12%	pT2/pT3a (62.5%/37.5%)	0%	Excluded	> 0.2 ng/ml	PSA, Stage, TV, Gleason
Lian et al. [8]	NSM v Apex. R	416	2.25	Open/lap (20%/80%)	< 8/ ≥ 8 (59%/41%	pT2/pT3 (65%/35%)	0%	Excluded	> 0.2 ng/ml	PSA, stage, Gleason, BMI, PSM, Perineural invasion
Pagono et al. [38]	NSM v Apex/Base, R	188/4734	2.23	Open	< 7/8/9–10 50%/20%/30%	T1/T2/T3 (53.8%/41%/4%)	12.2%—Adjusted	Excluded	> 0.2 ng/ml	A, race, PV, stage, PSA, Gleason, LN, Stage, PSM, Multifocal
Pierorazio et al. [39]	NSM v Base, R	79/16782	8	Robot/retropubic/Lap/perineal (4.2%/90%/5.6%/0.1%)	6/7/8–10 (60.7%/32.8%/6.5%)	T2/3a/3b (63%/33%/4%)	2.5%	6.3%	> 0.2 ng/ml	A, PSA, Gleason, Stage, Bladder neck margin
Vrang et al. [40]	NSM v Apex, R	214/605	2.7	Robot/Open (39/566)	6/7/8–10 (15.7%/76.2%/6.1%)	pT2/pT3/pT4 (75.4%/24%/0.3%)	1%—Excluded	Excluded	> 0.2 ng/ml	A, stage, PSA, Gleason, PSM
Wadhwa et al. 2016	NSM v Apex, R	142/4031	6.4	Retopubic/Pernieal/Lap/Robotic/NA (60%/11%/2%/17%/9%)	6/7/8–10 (41%/37%/14%)	pT1 + 2/pT3 + T4 (60%/40%)	0%	6%	> 0.2 ng/ml	A, stage, Gleason, year, PSA
Sofer et al. [17]	Apex vs Base, Ant, Post, Posterolateral, R	210/734	1.8	Open	6/7/8–10 (31%/41%/27%)	pT1/T2/T3 (60%/39%/1%)	0%	17% Adjusted in analysis	2 readings > 0.2 ng/ml	A, PSA, Gleason, Stage, T3a, TV, NS, Neoadjuvant
Obek et al. [18]	Apex vs Base, R	495	2.1	Open	6/7/8–10 (28%/42%/30%)	pT1/T2/T3 (53%/44%/3%)	6%	24.5% Neoadjuvant, adjusted in analysis	> 0.2 ng/ml	A, Gleason tumour location
Wu et al. [20]	Apex v Base/Anterior/Posterior, R	391	12.6	Open/LAP (84.4%/15.6%)	GG1/GG2/GG3/ > GG4 (40.6%/36.1%/9.2%/14.1%)	pT2/pT3 (67.6%/32.4%)	0%	Excluded	> 0.2 ng/ml	A, PSA, P size, Year, gleason, PSM, focality
Buschemeyer et al. [41]	Base vs NSM, R	79/1643	4.25 ± 3.5 (mean)	open	GG1/GG2/GG3-5 (19%/39%/42%)	pT1/T2 + T3 (54%/46%)	0%	Excluded	> 0.2 ng/ml	A, PSA, Race, Year, Centre, biopsy Gleason, Gleason, stage, Prostate weight
Kumano et al. [42]	NSM vs Apex, anterior, posterior, base, R	57/159	3.2	Lap	GS 7/7/8–10 (10.5%/84.2%/5.3%)	pT2/pT3a/pT3b (56.1%/29.8%/14.1%)	pN1 2.6%	Excluded	> 0.2 ng/ml	PSA, PSM, Gleason, Stage
Roder et al. [43]	NSM vs Apical, R	148/983	3.6 yr	Open/Robot (87.1%/12.9%)	GG1/GG2/GG3/GG > 4 (34.2%/50.9%/12%/2.4%)	pT2ab/c (14.4%/85.6%)	NA	Excluded	> 0.2 ng/ml	A, stage, Gleason, PSA, surgeon, Surgery type
Dash et al. [44]	NSM vs Base, R	60/1123	2.4	Open	G6/G7/ > G7 (20%/65%/12%)	T2 only	0%	Excluded	> 0.2 ng/ml	PSA, Gleason, stage, PSM, tumour size
Sasaki et al. [45]	NSM vs Apex, Middle, base, multifocal, R	798/2667	2.1	Robot	6/7/ > 8 (7.5%/72%/20.5%)	T2/T3a/b/T4 (71%/20.5%/8.5%/6%)	pN1 3.5%	Excluded	> 0.2 ng/ml	PSA, Gleason, Stage, NS, LN
Kurose et al. [21]	Apex vs Base, R	112/241	6	OPEN	6/3 + 4/4 + 3/ > 8 (5.7%/44.3%/30.3%/19.7%)	T2/T3a/b (62.3%/25.4%/12.3%)	6.6%	Excluded	> 0.2 ng/ml	PSA, Gleason, Stage, GS of tumour at margin

*Key–NSM* negative surgical margin, *GG* Gleason Grade group, *R* Retrospective Cohort study, *A* age, *PS* Prostate size, *TV* tumour volume, *LN* Lymph node status, *NS* nerve spare, *Gleason* Gleason score

### Study eligibility

The review considered all published studies, including reviews, randomized controlled trials, observational cohort studies and case-controlled studies. The language of publication was restricted to English. Rayyan QI (Covidence systematic review software, Veritas Health Innovation, Melbourne, Australia) and EndNote X8.2 (Clarivate Analytics, Philadephia, USA) were used to track studies included and excluded from the review.

### Statistical analysis

Multivariable Cox proportional hazard modelling coefficients (in the form of hazard ratios) for BCR (defined as PSA > 0.2 ng/ml post radical prostatectomy) were extracted where results had been adjusted for preoperative PSA, Gleason score and stage. Studies were subdivided based on the location of PSM used for comparison. The extracted information was tabulated according to pre-determined templates specified in our protocol (PROSPERO: CRD42022119025). Network meta-analysis was performed in the following subgroups: NSM vs apical PSM, bladder base PSM, posterolateral PSM, posterior PSM or anterior PSM for all techniques, with further subgroup analysis involving robotic and open techniques. The heterogeneity of the selected studies was calculated using the I^2^ score and Q statistics. A frequentist network meta-analysis with random effects was performed using R (packages used “netmeta”, “dmetar” and “dplyr”) [12–15]. Inconsistencies in the network model were identified using a net heat plot.

### Assessment of bias

Since all studies included in our systematic review were observational, the Newcastle Ottawa Scale for non-randomised studies was used to evaluate the risk of bias [16]. The scale was scored by two authors (AJ and JV). Publication bias was assessed using visual inspection of funnel plots where there were 10 or more studies present.

## Results

### Characteristics of included studies

**Fig. 1:**
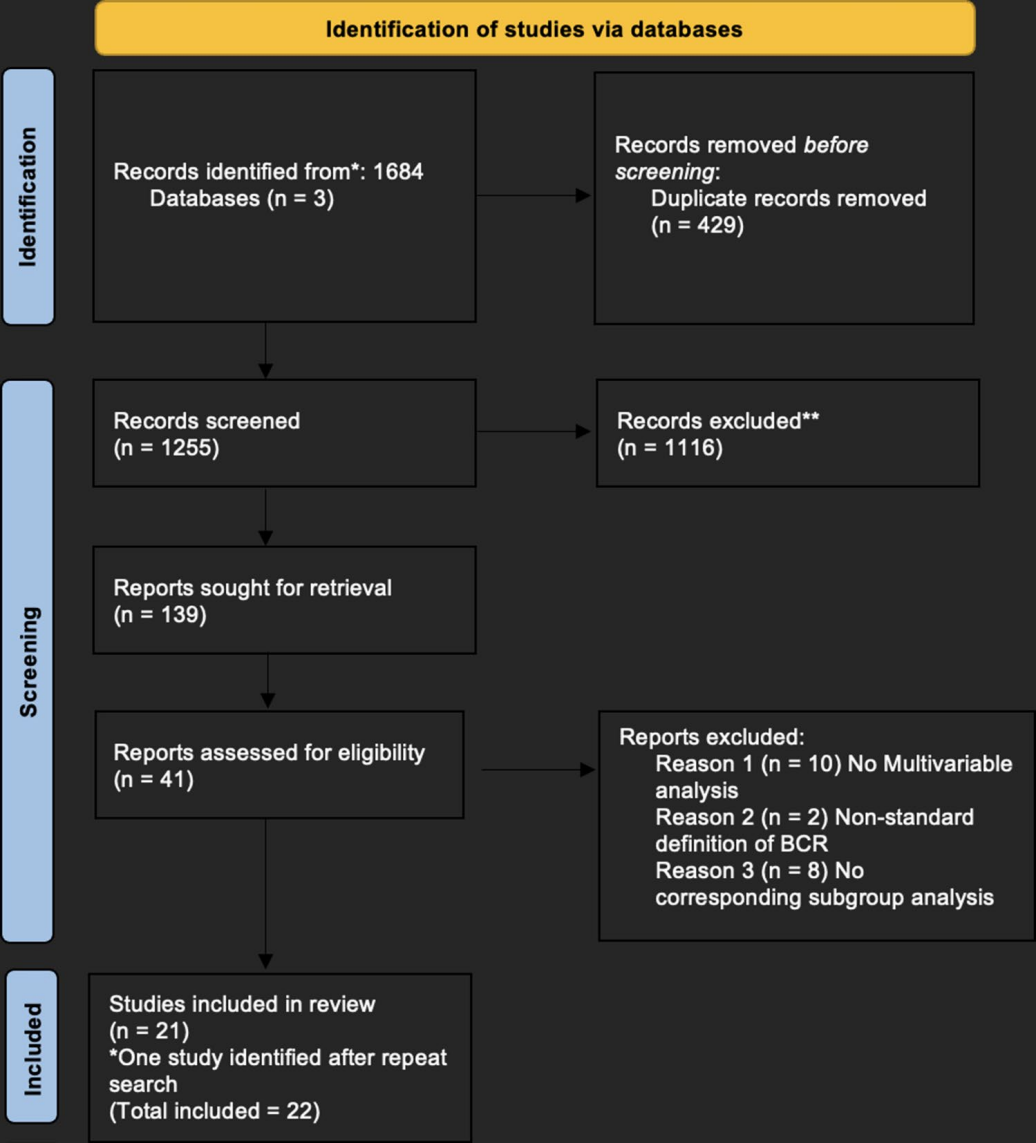
Preferred reporting items for systematic reviews and meta-analyses (PRISMA) flow chart of methodology

The search strategy identified 1255 studies across the MEDLINE, Scopus, Embase and Cochrane databases (Fig. 1). After exclusion of duplicate and irrelevant studies, 139 full-text articles were identified. 41 studies were identified after full text review. Of these 41 studies, 10 did not report a Cox multivariable hazards ratio and two were excluded due to a non-standard definition of BCR (PSA > 0.1 ng/ml). Eight studies met the criteria but could not be included in a corresponding subgroup analysis due to non-standard location definitions. The remaining twenty-two studies were included in the meta-analysis (Fig. 1).

A summary of included studies for meta-analysis can be found in Table 1. All studies were retrospective cohort studies published between 1999 and 2023 and most were from a single centre. The median/mean follow-up period ranged from 1.8–12.6 years. PSM sample size ranged from 117 to 798 patients. Surgical techniques varied between the use of robot-assisted, laparoscopic, and open radical prostatectomy.

There was significant variability in the comparison between surgical margin locations. Most studies performed comparisons between negative surgical margins (NSM) and other locations of PSM (apex, bladder base, posterior-lateral, anterior, and posterior). Five studies compared apical PSM with other locations [17–21], one study compared bladder base PSM with other locations [9] and one study compared posterolateral PSM with other locations [22] (Table 1).

### Overall results

**Fig. 2:**
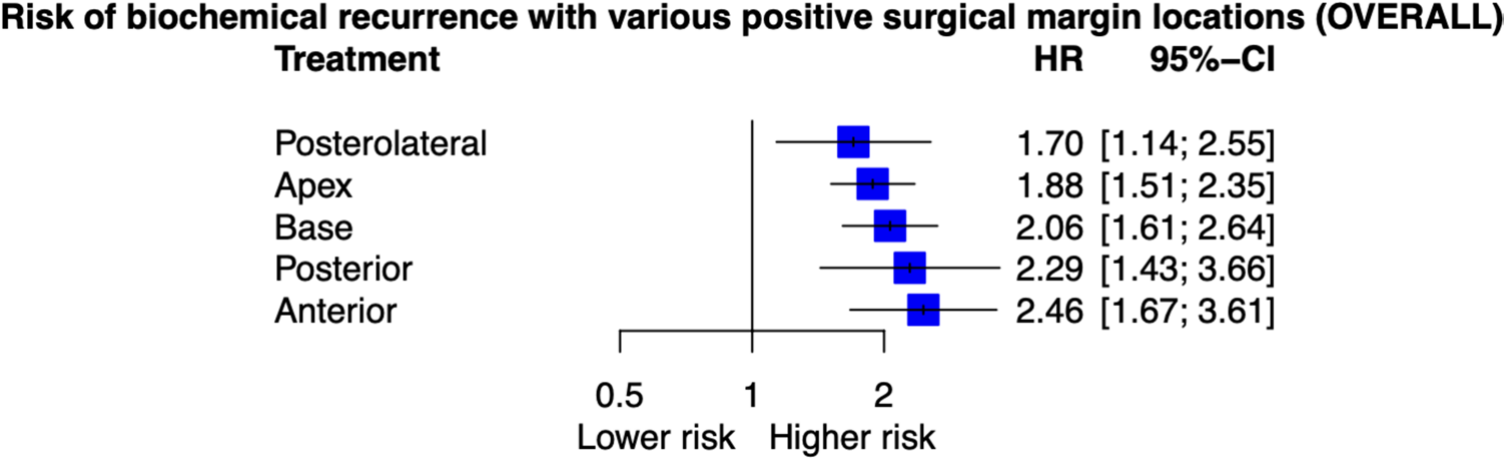
Network meta-analysis results comparing of biochemical recurrence in Various PSM locations compared to NSM for all studies

The comparisons exploring the risk of BCR between various PSM locations and NSM were made using both direct evidence and indirect evidence obtained from network meta-analysis. When comparing the risk of BCR to that of NSM patients, the anterior PSM conferred the highest risk (Direct evidence (DE): five studies, Hazard Ratio (HR) 2.46 (95% confidence interval (CI) 1.67–3.61), I^2^ = 76%, p < 0.01), followed by posterior PSM (DE: 3 studies, HR 2.29 (95% CI 1.43–3.66), I^2^ = 0%, p < 0.01), bladder base PSM (DE 12 studies, HR 2.06 (95% CI 1.61–2.64, p < 0.01), I^2^ = 69%), apical PSM (DE: 13 Studies HR 1.88 (95% CI 1.51–2.35), I^2^ = 59%, p < 0.01) and posterolateral PSM (DE: three studies, HR 1.70 (95% CI 1.14–2.25), I^2^ = 60%, p = 0.01) (Fig. 2). There were no inter-location comparisons that demonstrated a statistically significant difference in BCR risk. There was significant heterogeneity between studies used in overall analysis. The analysis revealed moderate to high heterogeneity in treatment effects across studies (I^2^ = 61%, 95% CI: 46.7% to 71.4%), significant overall variability (Q = 122.95, p < 0.0001), substantial within-design heterogeneity (Q = 108.58, p < 0.0001), and marginal inconsistency between designs (Q = 14.37, p = 0.0449).

### Subgroup analysis of robot-assisted laparoscopic prostatectomy

**Fig. 3:**
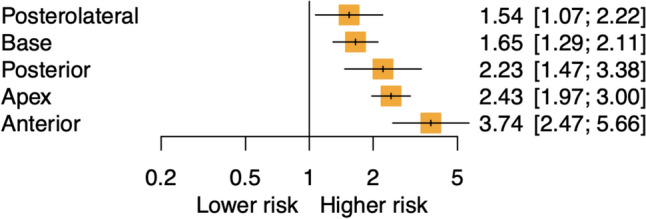
Subgroup network meta-analysis demonstrating BCR risk of various PSM for RALP

Sub-group analysis was performed to explore possible sources of heterogeneity including a comparison of robot-assisted laparoscopic prostatectomy (RALP) and open radical prostatectomy (ORP). In studies only including RALP, anterior PSM had the highest risk of BCR compared to NSM (DE: two studies, HR 3.74(95% CI 2.47–5.66) I^2^ = 0%, p < 0.01), followed by apical (DE: three studies, HR 2.43 (CI 1.97–8.00) I^2^ = 0%, p < 0.01), posterior (DE: one study, HR 2.23 (95% CI 1.47–3.38), p < 0.01), bladder base (DE: three studies, HR 1.65 (CI 1.29–2.11) I^2^ = 0%, p < 0.01) and posterolateral PSM (DE: one study, HR 1.54 (95% CI 1.07–2.22), p = 0.02) (Fig. 2B). For inter-location comparisons, anterior PSM demonstrated a higher risk of BCR compared to base (DE: one study, HR 2.27 (95% CI 1.45–3.54)) and posterolateral (DE: one study, HR 2.43 (95% CI 1.48–3.98)). Apical PSM demonstrated a higher BCR risk compared to base (DE: one study, HR 1.47 (95% CI 1.09–1.99) and posterolateral margin (DE: one study, HR 1.58 (95% CI 1.06–2.34) (Fig. 3A). This subgroup exhibited minimal heterogeneity, suggesting that stratification by surgery type may have addressed a significant source of heterogeneity in the primary analysis (I^2^ = 0%, Q total = 9.78, p = 0.6; Q between designs = 5.08, p = 0.4; Q within designs = 4.7, p = 0.6). The p-values for both Q total (p = 0.6) and Q between designs (p = 0.4) exceed 0.05, indicating no significant inconsistency between the direct and indirect comparisons in the network. There was an insufficient number of studies to perform a satisfactory subgroup analysis focusing on open radical prostatectomy patients.

### Assessment of bias

The studies conducted were non-randomized and retrospective, which means there may be selection bias. The outcomes can be biased due to inadequate blinding of histology reviewers. Most studies were classified as average to good quality based on the Newcastle Ottawa Scale, scoring lower if having inadequate follow-up time (< 5 years) (Supplementary Table 4). This needs to be considered when interpreting results, particularly for ORP subgroup analysis. Most studies included in the meta-analysis adjusted for Gleason score, preoperative PSA and stage during multivariable analysis. Funnel plot analysis suggested minimal publication bias (Supplementary Fig. 3).

## Discussion

**Fig. 4:**
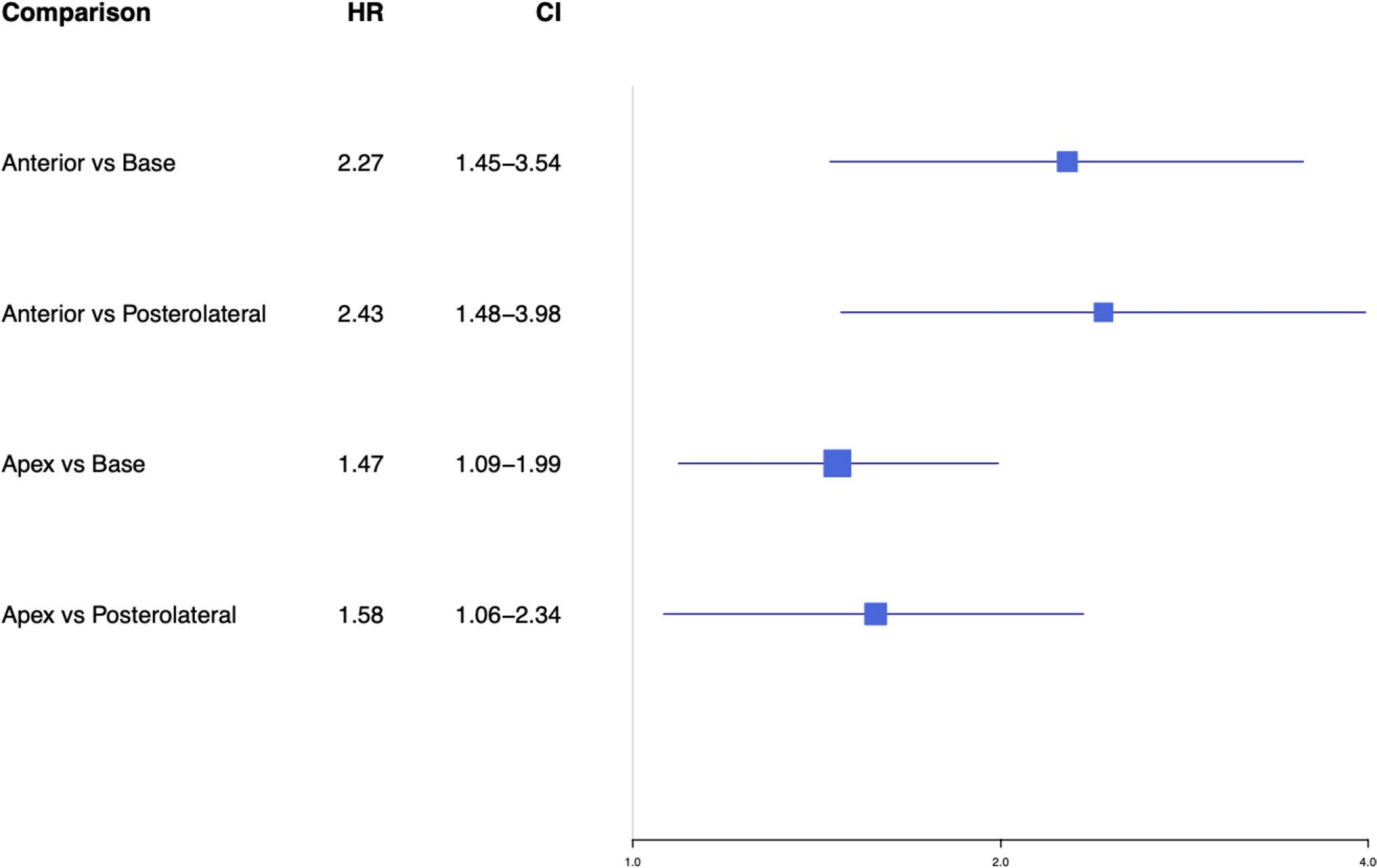
Clinically significant comparison of the risk of BCR between various inter-location comparison for studies including RALP

In this network meta-analysis, we provide evidence that the location of PSM influences the risk of BCR. More specifically, our primary analysis demonstrated that anterior PSM had the highest risk of BCR compared to NSM. Therefore, we recommend incorporating PSM location into future models to help categorize patients into a higher or lower risk of BCR. In the high-risk group, this may facilitate patient counselling and initiation of adjuvant treatment to achieve optimal oncological outcomes. In the low-risk group, it may potentially spare adjuvant treatment and prevent deterioration of functional outcomes [23]. Currently, adjuvant therapy is considered for high-risk patients with at least two out of three high-risk features (Gleason grade group 4 or 5, Pathological stage 3 and positive surgical margins) [24]. The rationale for this is based on the low numbers of PSM patients in key trials like RADICALs, which compared salvage and adjuvant radiotherapy. As a result, it was underpowered to demonstrate a clear advantage of salvage radiotherapy for patients with these high-risk features [25]. The use of adjuvant therapy in these patients with high-risk features is also supported by subsequent cohort studies[26]. Based on our findings, we encourage institutions to consider PSM location when counselling and determining surveillance or adjuvant treatments for these patients (Fig. 4).

In our primary analysis, we demonstrated that anterior PSM demonstrated the highest risk of BCR compared to NSM. This was followed by the posterior, base, apex and posterolateral PSM respectively. Various factors could have contributed to these findings. Anterior PSM may be indicative of anterior lobe prostate tumours, which are less common and often have a large tumour volume [7, 19]. This possibility also raises the issue of tumour volume being a confounder in our analyses, since very few studies have performed multivariable analysis to adjust for it. Hsu et al. propose that residual cancer in the anterior region may be more likely to proliferate due to its close proximity to the vascular supply, potentially leading to a higher risk of BCR [19]. There were insufficient studies to determine a statistically significant difference between the anterior, apex and posterior PSM. Posterior PSM may also represent aggressive disease compared to posterolateral and bladder base, although this was not formally demonstrated in our analysis. It has been suggested that the posterior fibrous capsule of the prostate, being dense and fused with Denonvilliers’ fascia, may be more resistant to tumour invasion. Consequently, tumours that do invade this fascia may be more likely to represent aggressive disease [7, 22].

The reliability and clinical interpretation of the primary analysis is challenging given the substantial heterogeneity of analysis, hence our decision to investigate the causes of heterogeneity using a RALP subgroup analysis and thus reflecting contemporary practice. In data relating to RALP, statistical heterogeneity was reduced to I^2^ = 0, indicating that surgical technique may contribute to variation. In the RALP subgroup, an anterior PSM had the highest risk of BCR followed by apical, posterior, bladder base and posterolateral PSM. The key difference when performing subgroup analysis was the increased relevance of apical margin in robotic subgroup analysis. Sooriakumaran et al. proposed that traction of the prostate during RALPs may account for this finding [27]. More specifically, traction caused by the non-dominant robotic arm enables a more magnified view to facilitate closer dissection to the prostatic capsule; this increases the risk of iatrogenic intra-capsular incision, which increases the risk of prostatic inked margin being in contact with the tumour and hence being deemed PSM [7]. The prostatic apex is an area that is very challenging to access and dissect, being intermingled with vital structures such as the dorsal venous complex, erectile nerves, rectum, and sphincter. The prostatic apex is at risk of intraprostatic incision given the desire to conserve urethral length for enhanced preservation of sexual and urinary function. RALP tends to improve visualisation and facilitate the dissection of this region. Apical margins are also more susceptible to invasion of cancer cells given the proximity to an abundant vascular supply that is more likely to be disrupted in ORP compared to RALP. Traditionally, apical margins were thought to be less relevant. Several studies in ORP series failed to demonstrate apical PSM as an independent predictor of BCR [7] though most of these studies had follow up period of less than five years. Based on our analysis, it appears that apical PSMs remain relevant in RALP.

This is the first systematic review to explore the location of PSM and its effect on BCR. There are several other systematic reviews investigating features of PSM after RP. For example, a recent meta-analysis exploring the role of the primary Gleason grade and Gleason grade group of the PSM on BCR demonstrated that Gleason grade > 3 at the PSM and increasing Gleason grade group of PSM is independently associated with a higher risk of BCR [28]. We have also published another systematic review demonstrating an increased risk of BCR associated with increasing PSM length [29]. Yossepowitch et al. performed a systematic review in 2014 exploring outcomes associated with PSM after radical prostatectomy [30] and alluded to studies that demonstrated the prognostic implications of the location of PSM, however, outcomes were not explored in detail with meta-analysis. A narrative review was also performed by Fontenot and Mansour, primarily as a method to standardise reporting styles of pathological parameters of positive surgical margin [31]. Our review improves upon these earlier studies and objectively explores the current evidence related to the location of PSM and its impact on BCR. Given the difference identified, further research should explore the outcomes of contemporary practices with greater adoption of the RALP and decreasing use of ORP.

This study has several strengths and weaknesses. We employed a network meta-analysis over a traditional pairwise meta-analysis, a strategy that increases the depth of results by extrapolating inter-location comparisons missing from studies. This offers a “bigger picture” analysis of results that otherwise would be fragmented using a single pairwise meta-analysis. The extrapolated estimates of indirect comparisons between various PSM location comparisons, which have not been performed in the literature, are useful given the rarity of positive surgical margins affecting these locations. However, the BCR risk comparisons between these locations of PSM still warrant formal testing to confirm our findings. Other strengths include only using studies that have performed multivariable analysis and thereby adjusting for covariates such as preoperative PSA and Gleason score, which are also known to increase the risk of BCR. The review also followed a protocol according to PRISMA guidelines and included atleast two different authors for screening, full-text review and data extraction. Limitations of this study include a low number of studies included for the subgroup meta-analyses and the use of retrospective cohort studies, meaning that the studies included may be prone to selection biases. This risk was assessed using the Newcastle Ottawa Scale with most studies receiving a score of 3 for the selection component (most points were lost for insufficient blinding and potential selection bias due to retrospective reviews). The overall heterogeneity between studies for the network meta-analysis was 61% which is substantial which makes overall analysis not as reliable with clinical interpretation compared to RALP subgroup analysis. The heterogeneity is accounted for by performing subgroup analysis which identified surgical technique (RALP, ORP, Perineal or Laparascopic) being the main contributor. Other contributors to heterogeneity include follow-up period, sample size, pathological features of included patients, use of lymph node-positive patients. We also note some studies included had less than five years of follow-up, which may not be sufficient to detect BCR events. Only a few studies adjusted for other pathological parameters of PSM such as Gleason score or grade of the PSM and PSM length. These are also important parameters that need to be considered when evaluating BCR risks as suggested by some studies, including our previous meta-analysis [28, 32, 33]. Another weakness is using BCR as the clinical endpoint; clinical progression by imaging would be a more relevant and reliable endpoint since it is a better surrogate marker of cancer-related mortality. Further longer-term studies exploring the relationship between positive surgical margin location and other outcomes such as systemic progression on imaging and cancer-related mortality are warranted, particularly considering the evolution of surgical practice.

## Conclusion

The location of positive surgical margins can affect the risk of BCR. Anterior PSM conveys the highest risk of recurrence with posterolateral margin being the least. Longer-term studies are needed to track other cancer endpoints like progression and mortality.

## Supplementary Information

Below is the link to the electronic supplementary material.

Supplementary file1 (DOCX 760 KB)

Supplementary file2 (DOCX 162 KB)

## Data Availability

The datasets generated during and/or analysed during the current study are available from the corresponding author on reasonable request.
