# Supplementary material for: Impact of positive surgical margin location after radical prostatectomy: a network meta-analysis
Source: World J Urol. 2025 Feb 22;43(1):134. doi: 10.1007/s00345-025-05479-7 (PMC11846713; doi:10.1007/s00345-025-05479-7)
Supplement: Supplementary file 1 — Supplementary file1 (DOCX 760 KB) [file 345_2025_5479_MOESM1_ESM.docx]

Appendix 1

Search terms for MEDLINE

Population:

Prostate cancer$ OR prostate neoplasm$ OR cancer$ of the prostate OR adenocarcinoma of the prostate OR prostatic cancer$ OR prostate gland cancer$ OR prostate tumour$ OR prostate adenocarcinoma$

AND

Intervention:

Radical prostatectomy OR prostatectomy OR prostate removal OR resection of prostate OR prostate surgery

AND

Comparators

(location OR PSM location OR APEX OR Bladder margin OR soft tissue margin) AND (positive surgical margin OR margin OR margin status OR PSM)

AND

Outcomes:

Oncological outcome$ OR Survival OR mortality OR metastases OR metastasis OR metastatic recurrence$ OR biochemical recurrence$ OR BCR OR biochemical failure$ OR biochemical relapse$ OR biochemical freedom from failure OR disease progression OR clinical recurrence OR clinical progression OR PSA failure OR PSA relapse OR PSA recurrence OR relapse free survival OR recurrence free survival OR local failure OR mortality rate OR Prostate specific antigen

Appendix 2

Figure 1: Network graph – All included studies


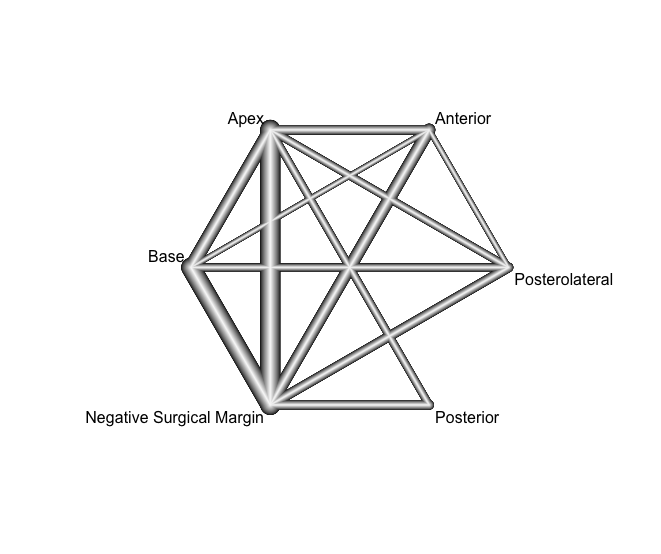


Figure 2 – Network graph – RALP only subgroup


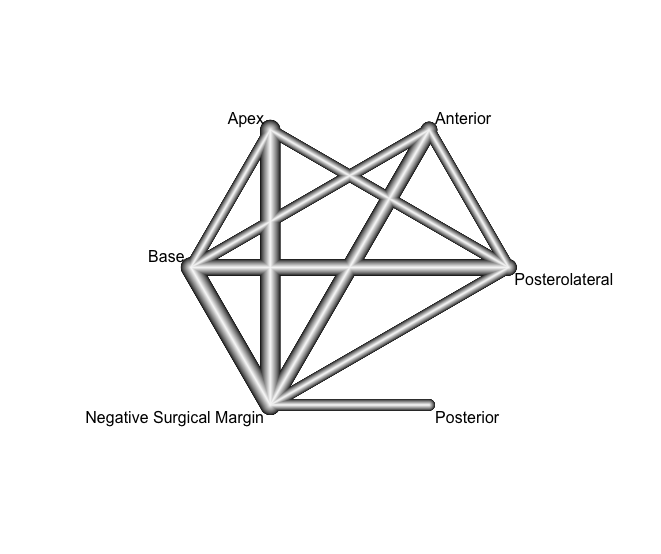


Appendix 3: Funnel Plot for all studies included


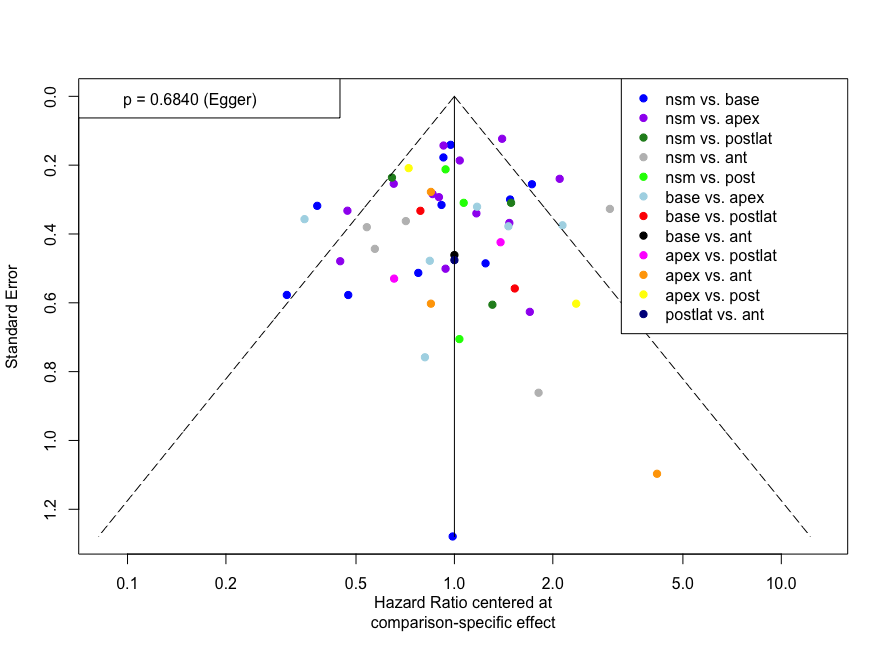


Appendix

Funnel plot demonstrating Interlocation comparison for all studies

Funnel plot demonstrating Inter-location comparison for Robot subgroup analysis

Appendix 4

Funnel plot demonstrating Inter-location comparison for Robot subgroup analysis


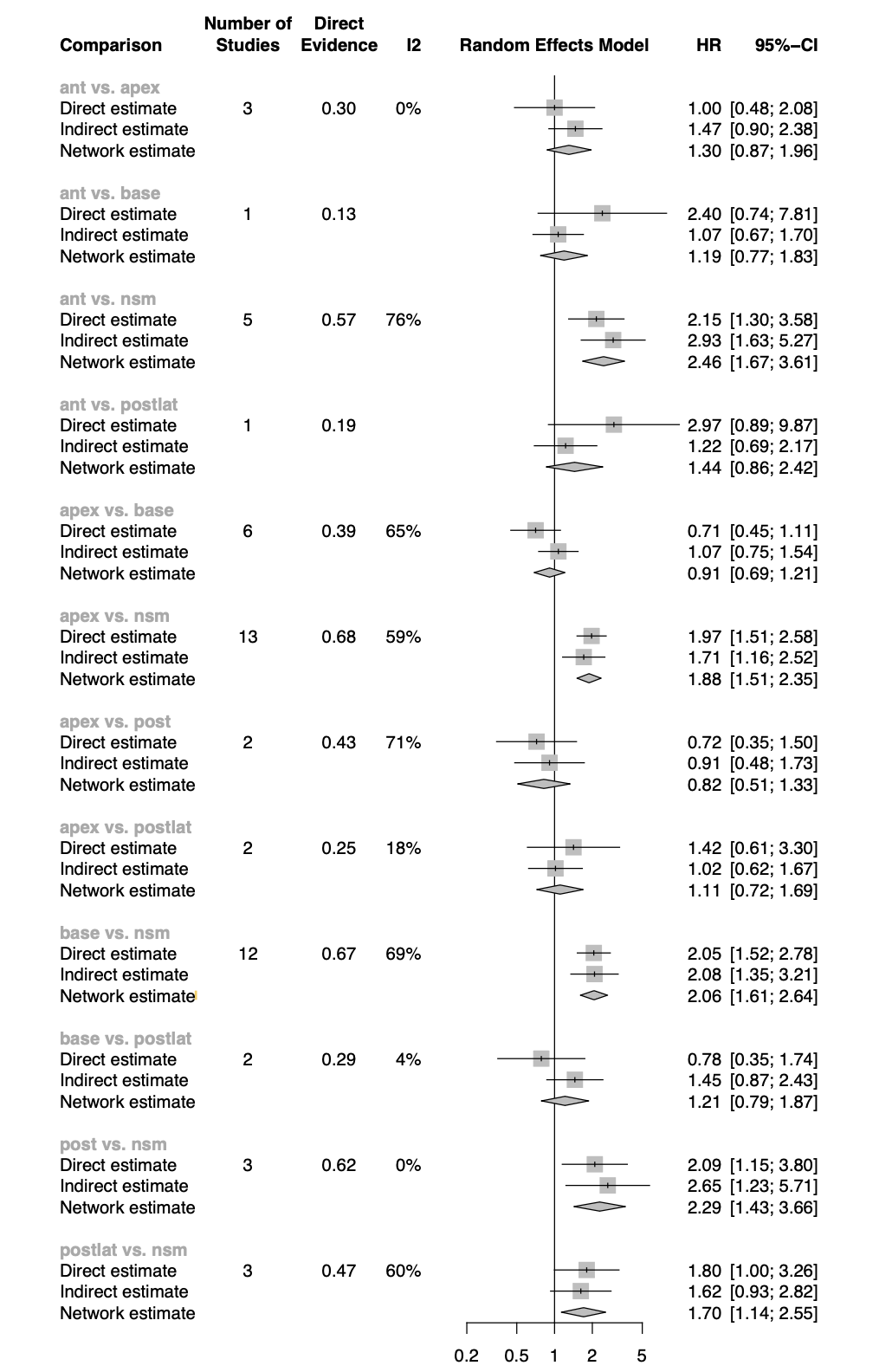


Appendix 5

Funnel plot demonstrating Inter-location comparison for Robot subgroup analysis


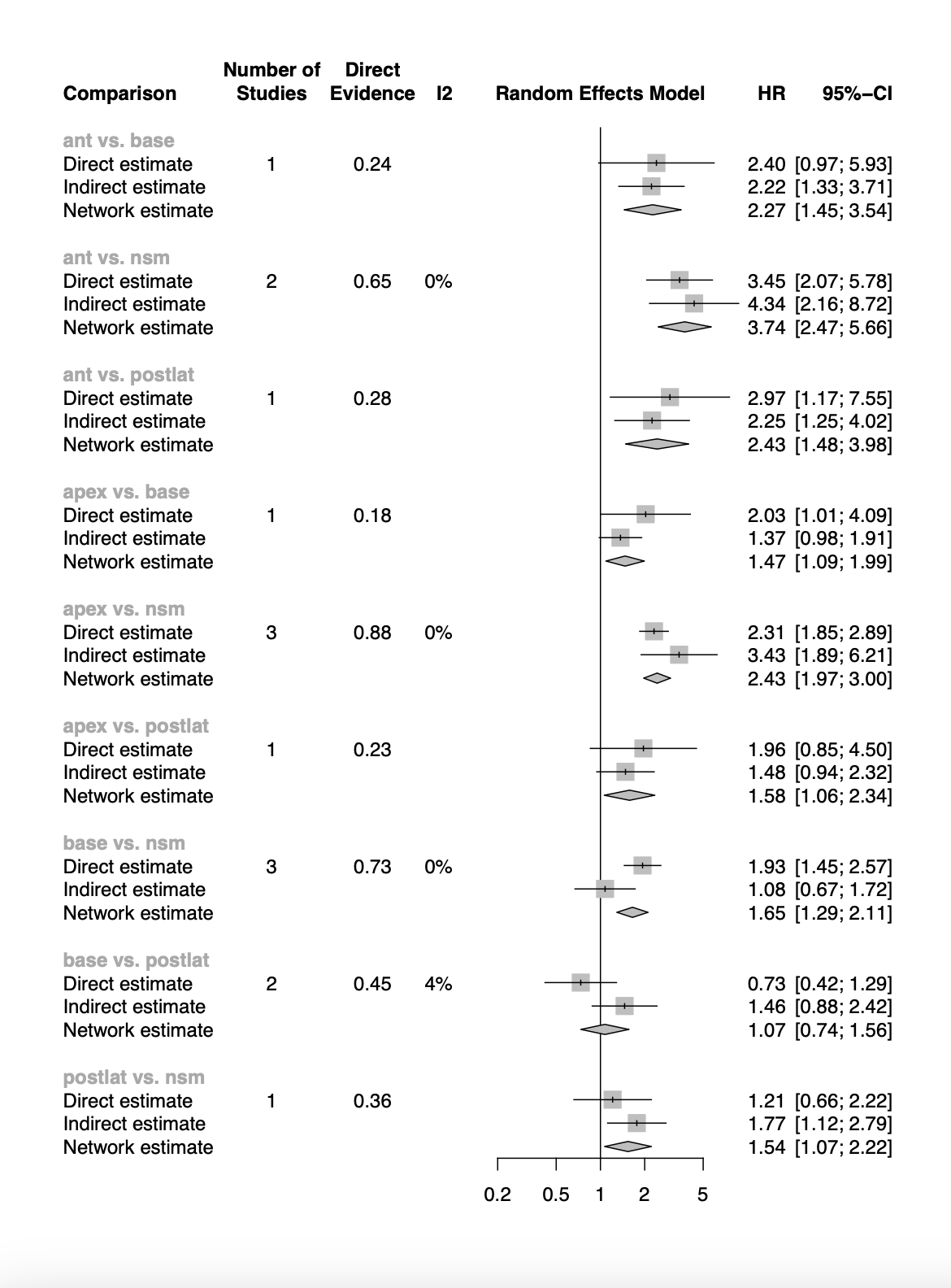


Appendix 6

Risk of bias – consensus

| **Study** | Selection | Comparability | Outcome | Score |
| --- | --- | --- | --- | --- |
| **Hsu et al. 2011** | **** | * | ** | Good |
| **Dev et al. 2015** | **** | ** | ** | Good |
| **Sammon et al2012** | **** | ** | ** | Good |
| **Sooriakumaranet. al 2014** | **** | ** | ** | Good |
| **Choo et 2013** | **** | ** | ** | Good |
| **Chung et al 2013** | **** | ** | * | Fair |
| **Eastham et al 2007** | **** | ** | ** | Good |
| **Kordan et al 2009** | **** | ** | * | Fair |
| **Lian et al 2020** | **** | ** | * | Fair |
| **Pagono et al 2016** | **** | ** | * | Fair |
| **Pierorazio et al 2010** | **** | ** | *** | Good |
| **Vrang et al 2012** | **** | ** | ** | Good |
| **Wadhwa et al 2016** | **** | ** | ** | Good |
| **Sofer et al 2002** | **** | ** | * | Fair |
| **Obek et al 1999** | **** | ** | ** | Good |
| **Wu et al 2018** | **** | ** | ** | Good |
| **Buschemeyer et al 2008** | **** | ** | ** | Good |
| **Kumano et al. 2008** | **** | ** | ** | Good |
| **Roder et al. 2014** | **** | ** | ** | Good |
| **Dash et al. 2002** | **** | ** | ** | Good |
